# Identification of C/EBPδ‐Modifying Compounds as Potential Anticancer Agents Using a High‐Throughput Drug Screen

**DOI:** 10.1111/jcmm.70287

**Published:** 2025-01-31

**Authors:** Leonie Hartl, JanWillem Duitman, Hella L. Aberson, Jan Paul Medema, Maarten F. Bijlsma, C. Arnold Spek

**Affiliations:** ^1^ Laboratory for Experimental Oncology and Radiobiology, Center for Experimental and Molecular Medicine Amsterdam UMC Location University of Amsterdam Amsterdam The Netherlands; ^2^ Cancer Biology and Immunology Cancer Center Amsterdam Amsterdam The Netherlands; ^3^ Department of Pulmonary Medicine Amsterdam UMC Location University of Amsterdam Amsterdam The Netherlands; ^4^ Department of Experimental Immunology Amsterdam UMC Location University of Amsterdam Amsterdam The Netherlands; ^5^ Inflammatory Diseases Amsterdam Infection & Immunity Amsterdam The Netherlands

**Keywords:** Akt, CCAAT/enhancer‐binding protein delta, cyclin‐dependent kinase, drug screen, mTOR, pancreatic ductal adenocarcinoma, PI3K

## Abstract

CCAAT/enhancer‐binding protein delta (C/EBPδ) has been shown to promote tumour growth, drug resistance and metastasis formation in some cancers, whereas we have shown that its re‐expression limits the features of tumour progression in pancreatic ductal adenocarcinoma (PDAC). The pharmacological targeting—either activation or inhibition—of C/EBPδ may therefore harbour clinical relevance and is desirable for preclinical studies on C/EBPδ in different contexts. Regrettably, to date, only few molecules have been identified that modify C/EBPδ. Here, we present a high‐throughput compound screen in conjunction with a novel eGFP reporter to identify further compounds that either increase or decrease C/EBPδ transcriptional activity. Of 1402 small molecule inhibitors, we identified a total of 22 potent inducers and 18 inhibitors of C/EBPδ‐mediated eGFP fluorescence. Using pathway enrichment analysis, we found that, generally, inhibition of the cell cycle elicits an increase in C/EBPδ activity whereas PI3K/Akt/mTOR‐targeting compounds reduce C/EBPδ activity. We confirmed the potential importance of cell cycle‐mediated regulation of C/EBPδ by showing that four of the most potent C/EBPδ activators—R547, PHA793387, AZD5438 and AT7519, all multi‐cyclin‐dependent kinase (CDK) inhibitors—limited the clonal expansion of PDAC cells. Next to providing a valuable selection of C/EBPδ‐modulating compounds for the use in preclinical studies, this report contributes to our understanding of the molecular regulatory mechanisms of C/EBPδ in general and in PDAC in particular.

## 1 Introduction

CCAAT/enhancer‐binding protein (C/EBP) delta is a member of the C/EBP family of transcription factors that plays a key role in tissue homeostasis and differentiation‐associated processes such as cell cycle regulation, proliferation and apoptosis [1, 2, 3]. As such, C/EBPδ has been shown to associate with patient survival in several cancers. In lung adenocarcinoma, glioma and urothelial bladder cancer, C/EBPδ is overexpressed and high C/EBPδ expression associates with poor survival [4, 5, 6]. A causal relationship between C/EBPδ and poor prognosis was suggested by showing that genetic ablation of *CEBPD* limits migration, invasion and proliferation in cell line models [4, 5, 6]. In contrast to the presumed tumour promotor role of C/EBPδ in several cancer types, C/EBPδ may actually limit tumour progression in other cancers. Indeed, C/EBPδ levels are diminished in hepatocellular carcinoma, pancreatic ductal adenocarcinoma (PDAC), ovarian cancer, breast cancer and cervical cancer, and low C/EBPδ levels associate with poor survival in these cancers [7, 8, 9, 10, 11, 12, 13, 14, 15]. As expected based on the presumed tumour suppressor role, re‐expression of C/EBPδ diminished the growth and/or migration of cancer cells in vitro [13, 14, 15]. Of note, C/EBPδ re‐expression in C/EBPδ low PANC‐1 and MIA PaCa‐2 PDAC cells reduced proliferation and clonogenic capacity, concurrent with decreased sphere formation capacity in soft agar assays [15].

Pharmacological targeting of C/EBPδ, either inhibition or re‐activation, depending on the tumour type, might thus be of (pre)clinical relevance. Problematically however, transcription factors other than those of the nuclear hormone receptor superfamily and the signal transducer and activator of transcription (STAT) protein family [16] are difficult to target. Unlike receptors or enzymes, most transcription factors lack an active site which can be targeted for inhibition or activation [17]. The lack of appropriate pharmacological C/EBPδ‐targeting compounds precludes further preclinical assessment of C/EBPδ as treatment modality in cancer and the actual clinical implications of C/EBPδ re‐expression remain elusive.

To advance our options for the preclinical targeting of C/EBPδ, we conducted a high‐throughput drug screen to identify small molecules with the ability to modify C/EBPδ activity. Due to the dichotomous role of C/EBPδ in cancer, we aimed to identify small molecules that may either *inhibit* or *activate* C/EBPδ activity. Instead of targeting C/EBPδ directly, the library applied here contains compounds that target specific proteins of well‐established pathways that potentially affect C/EBPδ transcriptional activity when targeted. Identifying compounds that indirectly alter the activity of C/EBPδ are not only valuable tools for those investigating C/EBPδ but also enhance our understanding of the mechanisms regulating C/EBPδ and are of potential relevance in diseases beyond cancer, where C/EBPδ is a known driver of disease progression.

## 2 Materials and Methods

### 2.1 Cell Lines and Cell Culture

Human embryonic kidney (HEK) 293 T cells (ATCC CRL‐321, Manassas, VA, USA) and human PANC‐1 (ATCC CRL‐1469) pancreatic cancer cell lines were maintained in Dulbecco's Modified Eagle Medium (#41965120, Gibco, Waltham, MA, USA) supplemented with 9% fetal bovine serum (FBS; #S‐FBS‐NL_015, Serana Europe GmbH, Pessin, Germany), 2% penicillin–streptomycin (#15140122, Gibco) and 2 mM L‐glutamine (#17‐605E, Lonza, Basel, Switzerland), hereafter referred to as complete growth medium, at subconfluence in a tissue culture incubator in 5% CO_2_ at 37°C. Cells were monthly tested negative for mycoplasma and their identity was confirmed yearly by STR profiling.

### 2.2 Cloning Strategy

Construction of the doxycycline‐inducible overexpression construct for C/EBPδ was described before [15]. To construct the C/EBPδ‐eGFP reporter construct, the C/EBPδ binding‐sequence 5′‐ATTGCAACAC‐3′ was adapted from Osada et al. [18] and concatenated thrice, separated by the restriction site sequences 5′‐GGATCC‐3′ for BamHI and 5′‐GCTAGC‐3′ for NheI. This sequence was cloned into the FpG5 vector (Addgene #69443, Watertown, MA, USA) between its BamHI and NheI restriction sites by GenScript (Leiden, the Netherlands). The complete sequence of the inserted DNA sequence is (with the C/EBPδ binding‐sequence underlined): 5′‐ggatccATTGCAACACggatccATTGCAACACgctagcATTGCAACACgctagc‐3′.

### 2.3 Lentivirus Production

The C/EBPδ‐eGFP reporter construct was transformed into competent cells (OneShot Top10 Chemically Competent *Escherichia* *coli* , Invitrogen #C404010, Thermo Fisher Scientific, Waltham, Massachusetts, USA) according to the supplier's protocol, then purified (NucleoSpin Plasmid EasyPure, Macherey‐Nagel, #740727, Düren, Germany) and validated by sequencing (BigDye Terminator, Applied Biosystems #4336697, Thermo Fisher Scientific). The resulting vector was incorporated in a third‐generation lentivirus system using pMDLg/pRRE (Addgene #12251), pRSV‐Rev (Addgene # 12253) and pMD2.G (Addgene #122) in HEK 293 T cells. Lentiviral particles were precipitated using PEG‐*it* virus precipitation solution (System Biosciences, #LV810A‐1, Sanbio, the Netherlands) according to the manufacturer's protocol.

### 2.4 Generation of Reporter Cell Line

HEK 293 T cells were transduced with the reporter construct in the presence of 8 μg/mL polybrene (Polybrene Infection/Transfection Reagent, Sigma‐Aldrich, #TR‐1003, Saint Louis, Missouri USA) and selected for efficient genomic integration using 0.25 mg/mL Hygromycin B (Sigma‐Aldrich #7772). Single clones were selected based on background eGFP‐fluorescence as relatively high baseline eGFP‐fluorescence allows the detection of both C/EBPδ‐suppressing as well as ‐activating agents. For the validation experiment, three clones were pooled, seeded in 96‐well plates, transduced with the doxycycline‐inducible C/EBPδ overexpression construct and treated with increasing concentrations of doxycycline (0, 0.001, 0.01, 0.1, 1.0, 2.0 and 10.0 μg/mL) for 72 h. Next, cells were trypsinised and resuspended in fluorescence‐activated cell sorting (FACS) buffer (1% FBS in PBS) and eGFP expression was quantified using the FITC‐A‐channel of the CytoFLEX S flow cytometer (Beckman Coulter, Brea, CA, USA).

### 2.5 Compound Screen

HEK 293 T C/EBPδ‐eGFP reporter cells (5 × 10^3^) were seeded in 96‐well plates in complete growth medium and left to attach overnight. The following day, 1402 compounds of the inhibitor library (#L1100, Selleckchem, Planegg, Germany) were diluted 100× in OptiMem (#31985062, Gibco) and added to the cells at a final concentration of 1 μM (1st exploration screen) or 0.01, 0.1 and 1 μM (2nd validation screen) for 72 h. Cells were then washed, detached with TripLE (#12604013, Gibco) and resuspended in 50 μL FACS buffer (1% FBS in PBS). Fluorescence intensity of eGFP was analysed using the CytoFLEX S flow cytometer (Beckman Coulter). eGFP expression of compound‐treated cells was normalised to DMSO‐treated control cells and analysed using FlowJo Software (BD Research Cloud, Ashland, OR, USA).

### 2.6 RT‐qPCR

To determine *CEBPD* gene expression levels, cells were lysed for RNA extraction using the NucleoSpin RNA‐extraction kit (#740955, Macherey‐Nagel) according to the manufacturer's protocol. RNA yields were quantified and analysed spectrophotometrically using the NanoDrop 2000 (#ND‐2000, Thermo Fisher Scientific), treated with RQ1 RNAse‐Free DNAse (#M6101, Promega, Madison, WI, USA) and reverse‐transcribed with M‐MLV Reverse Transcriptase (#M1701, Promega), random hexamers (#N8080127, Thermo Fisher Scientific) and 10 mM dNTPs (#R0192, Thermo Fisher Scientific). The SensiFAST SYBR No‐ROX Kit (#BIO‐980, GC biotech, Alphen aan den Rijn, the Netherlands) was used for real‐time quantitative RT‐qPCR using the LightCycler 480 Instrument (Roche, Woerden, the Netherlands). Gene expression levels were normalised to *TBP* and *RPLP0* expression using the primers listed in Table S1.

### 2.7 Clonogenic Assay

PANC‐1 cells (1–8 per well) were seeded into 96‐well plates in the presence of DMSO (negative control) or 0.5, 0.1 or 0.02 μM of the indicated compound for 3 weeks. Compounds were refreshed twice a week, and after 3 weeks, cells were fixed and stained using crystal violet (0.5% crystal violet in 6% glutaraldehyde) for 30 min at room temperature followed by three washes in tap water.

### 2.8 Statistical Analysis

Mean eGFP expression and standard deviations of the first compound FACS screen were calculated in Excel (Microsoft, Redmond, WA, USA). The fold enrichment of pathways was also calculated in Excel (Microsoft) and defined as the number of observed compounds/the number of expected compounds in which the number of expected hits is defined as the number of compounds in a specific pathway × (*y*/1402) where *y* is the number of compounds confirmed to modify C/EBPδ in the validation run and 1402 is the total number of compounds in the library. Significance was analysed using the chi‐squared function in Excel.

### 2.9 Illustrations

**FIGURE 1:**
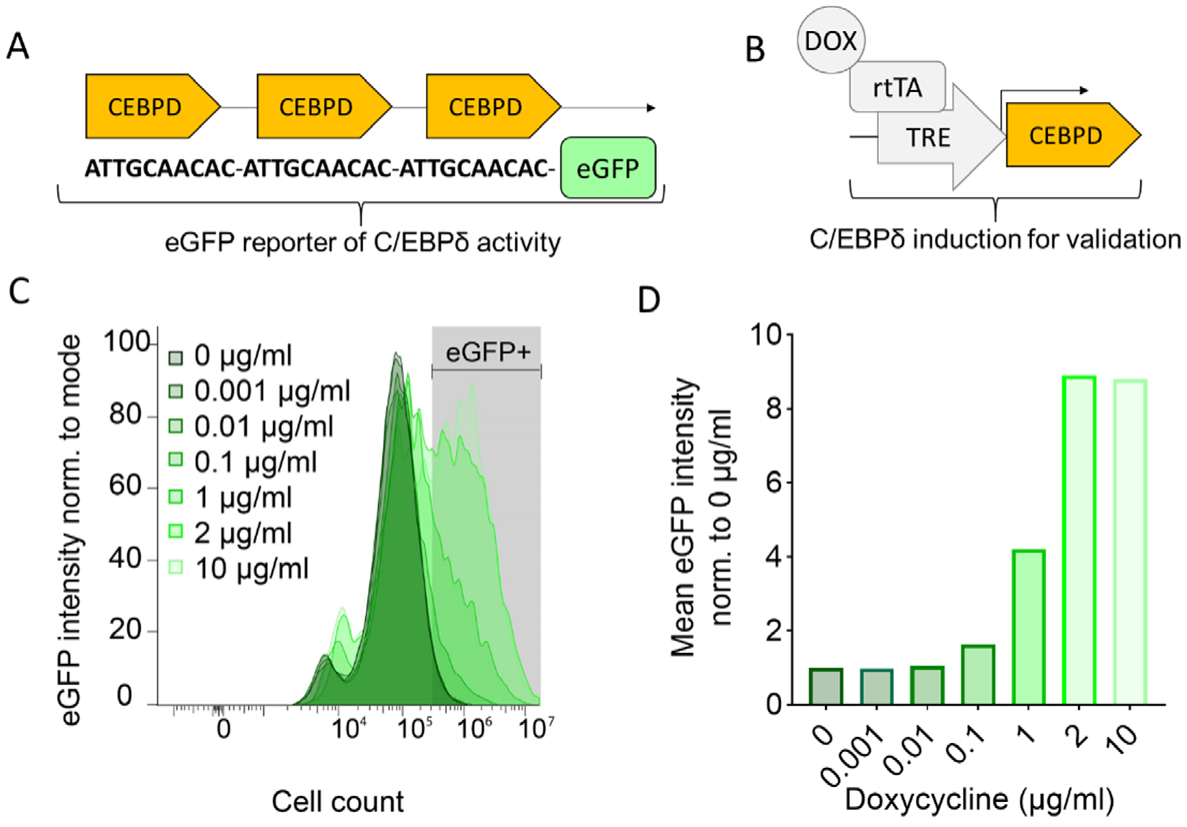
Construction and validation of a C/EBPδ‐eGFP‐reporter cell line in which GFP levels are directly proportional to the transcriptional activity of C/EBPδ. (A) The C/EBPδ‐eGFP reporter construct contains 3 C/EBPδ‐binding sites followed by the coding sequence for eGFP. (B) The validation construct contains a Tet‐on construct for induction of CEBPD transcription. DOX: Doxycycline, rtTA: Reverse tetracycline‐controlled transactivator, TRE: Tet‐response element. (C) To validate eGFP‐sensitivity, HEK 293 T cells transduced with both constructs, (A) and (B), were treated with the indicated concentration of doxycycline for 72 h and eGFP intensity was measured by FACS. In the respective conditions with increasing concentrations, 4.08%, 3.78%, 4.15%, 12.2%, 31.3%, 50.8% and 53.1% of single cells were eGFP positive. (D) Bars indicate the relative increase in eGFP intensity with increasing doxycycline concentration as derived from (C).

All graphs except Figure 1A–C were made in GraphPad Prism (version 9.3.1, GraphPad Software Inc., San Diego, CA, USA). Figure 1A,B were made using Power Point (Microsoft) and Figure 1C was made using FlowJo Software (BD Research Cloud).

## 3 Results

### 3.1 Construction and Validation of a C/EBPδ Reporter Cell Line

To identify compounds that modify (i.e., activate or inhibit) C/EBPδ activity, we first established a C/EBPδ‐eGFP reporter cell line in which GFP expression directly correlates to transcriptional activity of C/EBPδ. To this end, the C/EBPδ DNA‐binding sequence −*5′‐ATTGCAACAC‐3′*‐ [18] was concatenated thrice and cloned in front of the coding sequence for eGFP (Figure 1A). This C/EBPδ‐eGFP reporter construct was subsequently introduced into HEK 293 T cells. This commonly used cell line for heterologous protein expression shows low baseline C/EBPδ (and thus eGFP) levels, allowing to identify compounds that either enhance or decrease C/EBPδ activity. To validate the sensitivity of eGFP to C/EBPδ activity in the created reporter cell line, a subset of cells was transfected with a previously described doxycycline (DOX)‐inducible C/EBPδ overexpression plasmid which induces C/EBPδ transcription in a doxycycline dose‐dependent manner (Figure 1B) [15]. As expected, increasing concentrations of doxycycline and consequent C/EBPδ in the validation cell line led to an increase of eGFP fluorescence (Figure 1C). Indeed, DOX concentrations up to 0.1 μg/mL showed no or minimal eGFP induction but higher DOX concentrations induce eGFP levels by fourfold to maximal ninefold with 1 μg/mL and 2–10 μg/mL, respectively.

### 3.2 Reporter Assay to Identify Small Molecule Inhibitors That Strongly and Repeatedly Modify C/EBPδ Activity

**FIGURE 2:**
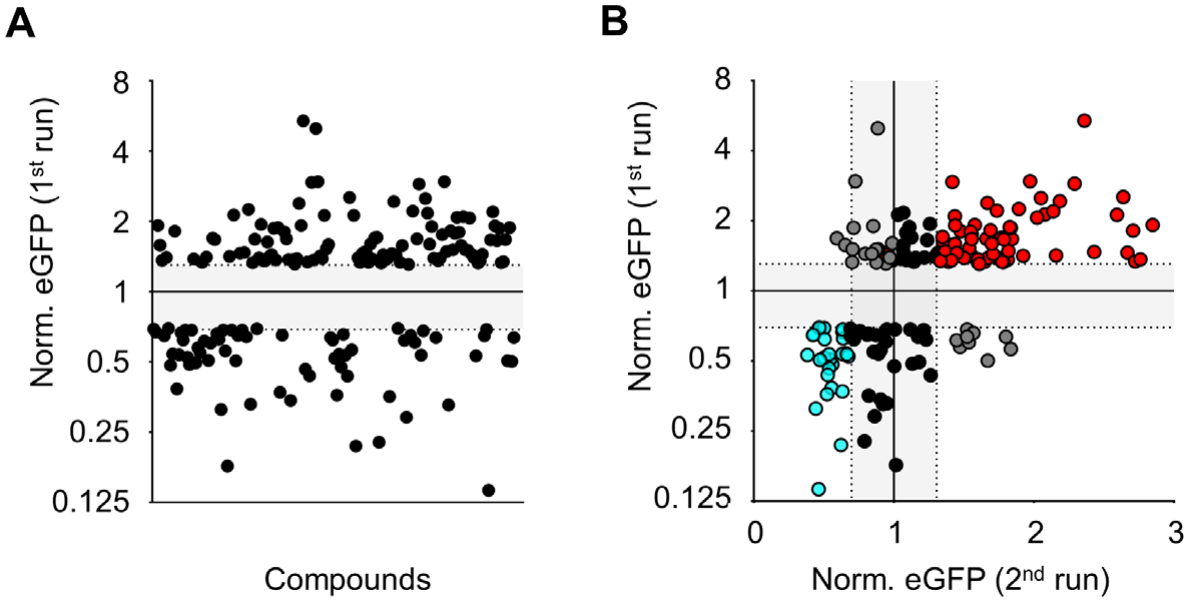
Selection of small molecule compounds that reproducibly modulate C/EBPδ‐induced eGFP expression. (A‐B) Full lines at *y* = 1 show the mean fluorescent intensity of all compound‐treated cells normalised to DMSO‐treated cells. Plotted are all compounds exceeding a fluorescent intensity of the mean ± 1 SD (dotted lines) in the first run (A) and the corresponding resulting eGFP expression in a second run combined with the data obtained from the first one (B). Red and blue marks represent compounds that reproducibly exceeded these thresholds, compounds marked black performed poorer in the second run and grey marks represent compounds that showed opposing effects in both runs.

To identify new compounds that modify C/EBPδ activity, we next applied a library of 1402 compounds to the C/EBPδ‐eGFP‐reporter cells (without the DOX‐inducible C/EBPδ overexpression plasmid). This compound library contains cell‐permeable small molecule inhibitors targeting specific signalling pathways [19]. All compounds were added at a final concentration of 1 μM (based on [20]) and FACS was used to determine the effect of each compound on C/EBPδ‐mediated eGFP signal intensity after a 72 h treatment. In a first run, we identified 105 individual compounds that increased eGFP intensity by more than one standard deviation (SD) above the mean after normalisation to DMSO‐treated controls (Figure 2A). Interestingly, we also found that 67 compounds had a suppressive effect on C/EBPδ activity (Figure 2A). All identified C/EBPδ‐modulating compounds were taken to a second (validation) run which confirmed the results of the first run (Pearson *r* = 0.52; *p* < 0.0001) and showed that of the 105 C/EBPδ‐inducing compounds, 83 again induced C/EBPδ activity (Figure 2B, red and black dots above *y* = 1), of which 65 repeatedly induced C/EBPδ activity towards ≥ 1 SD above the mean (Figure 2B, red dots above *y* = 1; Table S2). Also, of the 67 C/EBPδ‐suppressing compounds, 45 again suppressed C/EBPδ activity, of which 29 repeatedly suppressed the reporter signal to ≥ 1 SD below the mean eGFP intensity (Figure 2B, black, resp. blue dots below *y* = 1; Table S2).

### 3.3 Pathway Analysis to Identify Biological Targets That Modify C/EBPδ Activity

**FIGURE 3:**
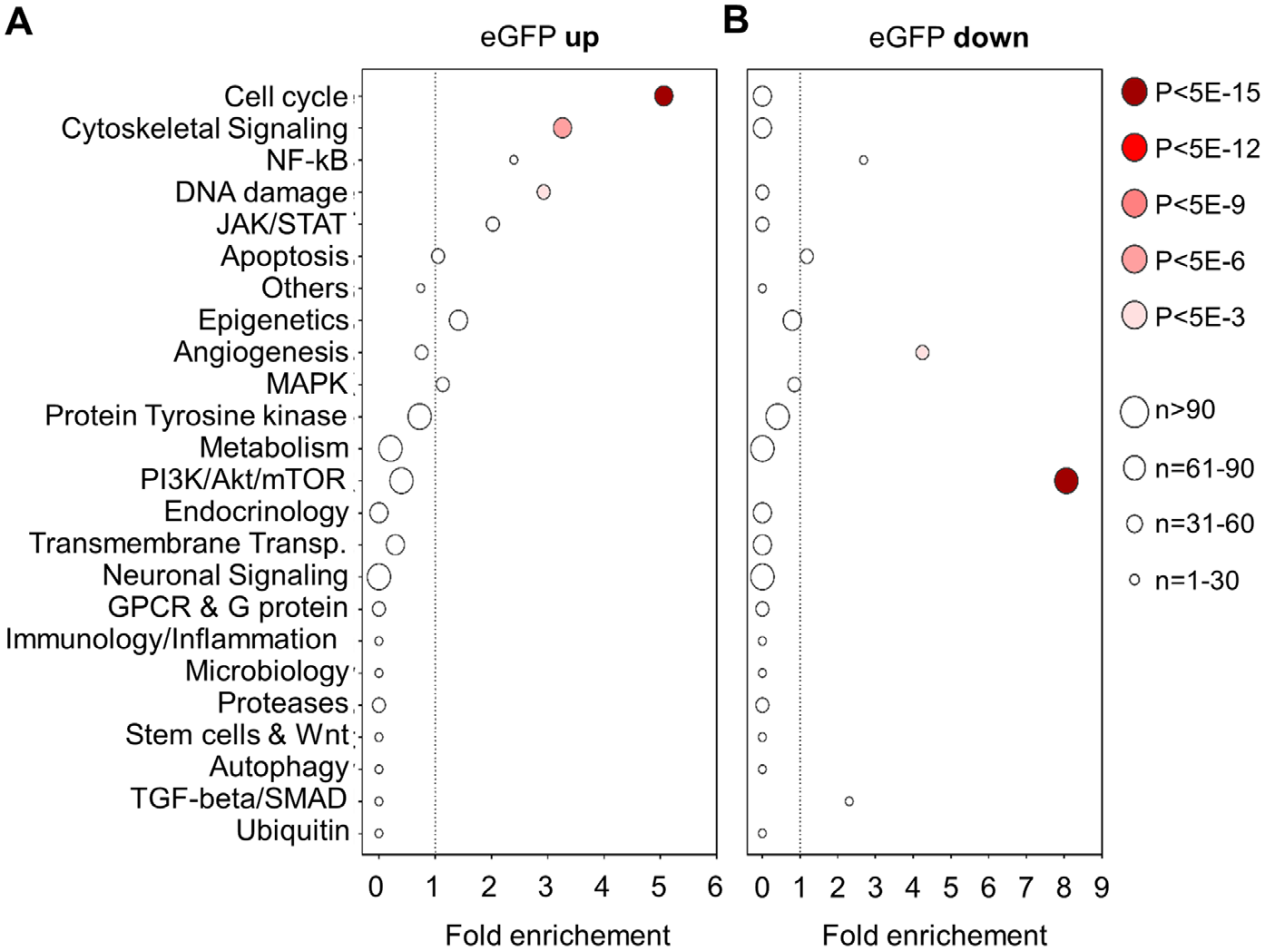
Pathway enrichment scores using compounds that consistently modify C/EBPδ activity. Targeted pathways are listed on the *y*‐axis, their enrichment score on the *x*‐axis. Panel (A) includes all compounds that upregulated eGFP‐expression, panel (B) includes all compounds that downregulated C/EBPδ‐mediated eGFP signal intensity. *n*, amount of compounds in the library that target the specific pathway.

The compounds contained in the applied library inhibit specific known pathways, respective of their biological target (Figure S1). To get insights into the pathways that upregulate C/EBPδ, we used the compounds that enhanced eGFP expression above the 1 SD threshold in both runs (red marks above *y* = 1, *n* = 65 in Figure 2B) to compute pathway enrichment scores. Pathways enriched among these compounds are potentially involved in the regulation of C/EBPδ. Figure 3A shows the most enriched pathways along with their enrichment scores, the significance of this enrichment compared to the expected distribution of pathways and the number of compounds falling into each pathway. This analysis suggests that the inhibition of cell cycle‐related targets most efficiently promote an activation of C/EBPδ in HEK 293 T cells, whereas cytoskeletal signalling and DNA damage inhibitors may also induce C/EBPδ activity although clearly to a lesser extent. To identify pathways that downregulate C/EBPδ, we next used the compounds that suppressed eGFP expression below the 1 SD threshold in both runs (blue marks below *y* = 1, *n* = 29 in Figure 2B). This analysis showed that inhibition of the PI3K/Akt/mTOR pathway has a strong suppressive effect on C/EBPδ activity, whereas angiogenesis inhibitors may also suppress C/EBPδ activity, although this pathway shows clearly less significant differences (Figure 3B).

### 3.4 CDK Inhibitors Induce C/EBPδ Activity and PI3K/AKT/mTOR Pathway Inhibitors Repress C/EBPδ Activity

To further enhance the reliability of our results, we included lower concentrations of each compound in the validation screen described above. In addition, we analysed cell numbers after compound treatment based on the notion that C/EBPδ induction in HEK 293 T does not induce cytotoxicity (Figure 1) implying that compounds killing these cells are cytotoxic irrespective of C/EBPδ levels. Therefore, compounds exhibiting severe cytotoxicity at their effective concentration were excluded from further analysis. This narrowed down the selection of the initial 65 compounds which strongly and reproducibly induced C/EBPδ to a final list of 22 candidates which exhibit no cytotoxicity in the tested HEK 293 T cells (red cells in Table 1). Next to this, 18 compounds reproducibly suppressed C/EBPδ activity without being cytotoxic towards HEK 293 T cells (blue cells in Table 1).

**TABLE 1:** Non‐cytotoxic compounds reproducibly modifying C/EBPδ to ≥ 1 SD above (red) or below (blue) the DMSO‐normalized mean.

Compound	Target	Pathway	1 μM^a^	0.1 μM	0.01 μM
(+)‐JQ1	BET	Epigenetics	2.31	2.04	1.46
AT7519	CDK	Cell Cycle	2.06	2.04	1.94
AZD5438	CDK	Cell Cycle	2.03	1.69	1.17
CHIR‐98014	GSK‐3	PI3K/Akt/mTOR	1.79	1.28	1.27
CPI‐203	Epigen. Reader Domain	Epigenetics	2.28	1.69	1.47
Digoxin	Sodium Channel	Transmemb. Transport	1.55	2.56	1.51
JNJ‐7706621	CDK, Aurora Kinase	Cell Cycle	1.76	1.01	1.10
M344	HDAC	Cytoskeletal Signaling	2.36	1.13	1.05
MK‐8745	Aurora Kinase	Cell Cycle	1.45	1.37	1.30
MLN8054	Aurora Kinase	Cell Cycle	1.49	1.37	1.35
OTX015	BET	Epigenetics	2.59	1.85	1.67
PD168393	EGFR	Protein Tyrosine Kinase	1.42	1.24	1.27
PHA‐793887	CDK	Cell Cycle	2.07	1.67	1.25
Pracinostat	HDAC	Cytoskeletal Signaling	2.11	1.43	1.23
R547	CDK	Cell Cycle	3.88	2.12	1.47
RAF265	Raf, VEGFR	MAPK	1.38	1.02	1.15
RITA	p53	Apoptosis	1.53	1.69	1.36
Stattic	STAT	JAK/STAT	1.38	1.26	1.26
Tenovin‐1	p53	Apoptosis	1.86	1.59	2.18
TH‐302	Others	Others	1.53	1.25	1.18
Triapine	DNA/RNA Synthesis	DNA Damage	1.76	1.38	1.39
Voreloxin	Topoisomerase	DNA Damage	1.66	1.18	1.46
AZD5363	Akt	PI3K/Akt/mTOR	0.49	0.74	1.15
Bosutinib	Src	Angiogenesis	0.58	0.93	0.92
Dasatinib	Src, Bcr‐Abl, c‐Kit	Angiogenesis	0.60	0.97	0.97
GDC‐0068	Akt	PI3K/Akt/mTOR	0.54	0.69	1.00
GDC‐0941	PI3K	PI3K/Akt/mTOR	0.65	1.03	1.23
GSK1059615	PI3K, mTOR	PI3K/Akt/mTOR	0.38	0.91	0.93
GSK690693	Akt	PI3K/Akt/mTOR	0.47	0.64	1.04
GZD824	Bcr‐Abl	Angiogenesis	0.58	0.54	0.77
INK 128	mTOR	PI3K/Akt/mTOR	0.44	0.42	0.49
KU‐0063794	mTOR	PI3K/Akt/mTOR	0.53	0.94	1.01
Pexmetinib	p38 MAPK	MAPK	0.69	0.89	0.91
Ponatinib	Bcr‐Abl, VEGFR	Angiogenesis	0.50	1.00	1.06
PP242	mTOR	PI3K/Akt/mTOR	0.60	1.09	1.09
Rapamycin	mTOR	PI3K/Akt/mTOR	0.59	0.63	0.72
SGC‐CBP30	Epigen. Reader Domain	Epigenetics	0.60	1.04	1.26
Temsirolimus	mTOR	PI3K/Akt/mTOR	0.60	0.64	0.76
WYE‐354	mTOR	PI3K/Akt/mTOR	0.63	0.97	0.99
ZSTK474	PI3K	PI3K/Akt/mTOR	0.60	1.00	1.27

^a^ Average eGFP level of run 1 and run 2.

**FIGURE 4:**
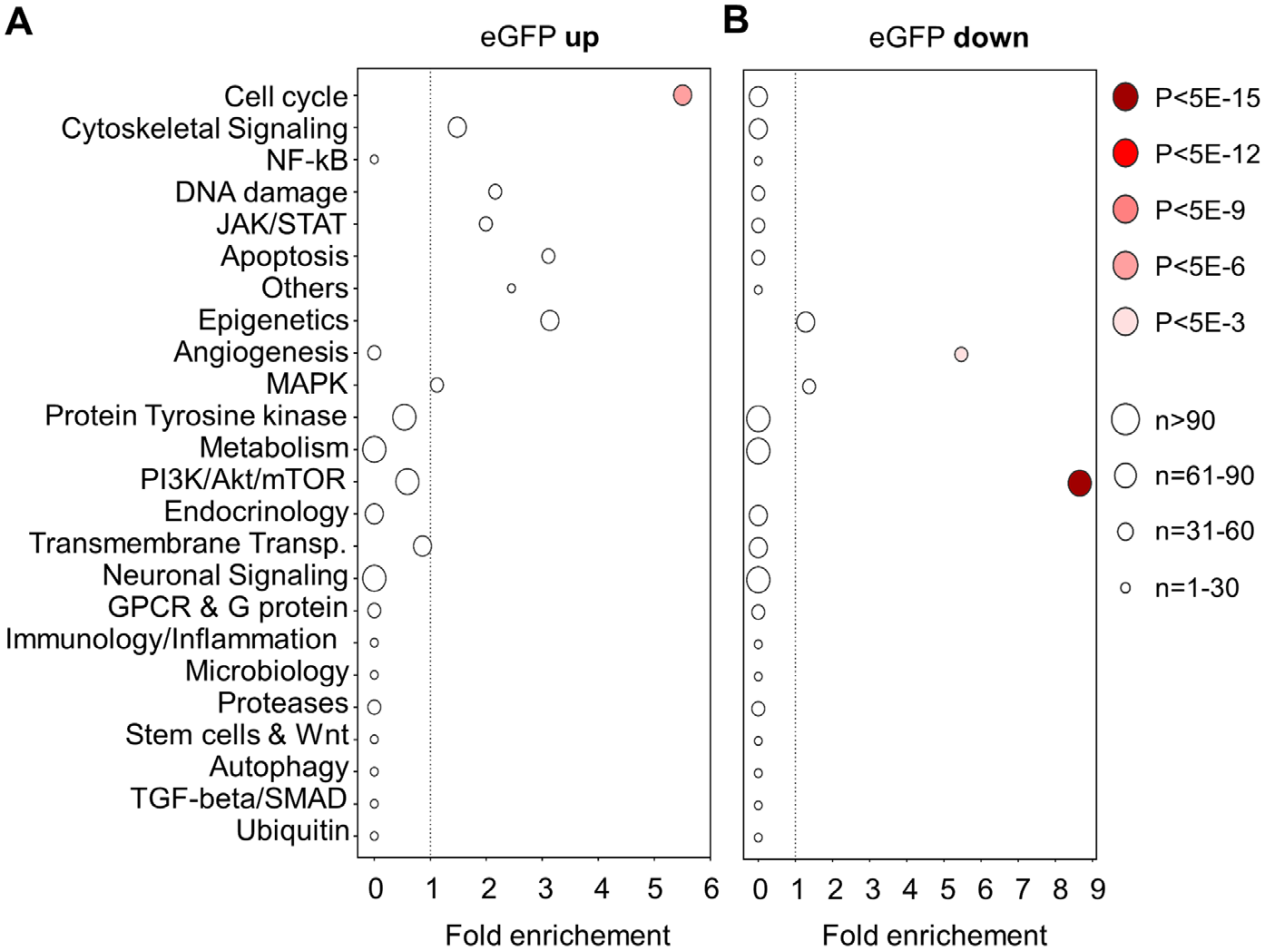
Pathway enrichment scores using all compounds that consistently modify C/EBPδ activity without inducing cytotoxicity. Targeted pathways are listed on the *y*‐axis, their enrichment score on the *x*‐axis. Panel (A) includes all compounds that upregulated eGFP‐expression, panel (B) includes compounds in the library that downregulated C/EBPδ‐mediated eGFP signal intensity. *n*, amount of compounds targeting a pathway.

Subsequent pathway analysis using the compounds listed in Table 1 confirms that compounds targeting the cell cycle are over‐represented among the 22 selected compounds that induce C/EBPδ expression whereas cytoskeletal and DNA damage targeting compounds lose their significance (Figure 4A). Notably, PI3K/AKT/mTOR inhibitors are highly and very significantly enriched among the 18 selected compounds that repress C/EBPδ expression (Figure 4B).

In the entire library, compounds annotated to affect the cell cycle target a rather broad range of proteins like, among others, CDKs, Aurora kinases, PLK, Rho and ROCK. Interestingly however, with regard to C/EBPδ induction, the enriched compounds in the cell cycle pathway exclusively target CDKs and/or Aurora kinases (Table 1, Table S3), leading to enrichment scores of 11.4 (*p* = 4.06E‐12) and 8.7 (*p* = 5.26E‐6) for CDK and Aurora kinase inhibitors, respectively. Among the different targets in the PI3K pathway that has been shown to associate with decreased C/EBPδ expression, only genuine PI3K/Akt/mTOR inhibitors are enriched among the 18 selected compounds, whereas other targets that make up the PI3K/Akt/mTOR pathway like AMPK, GST3 and S6 kinase are not enriched in our compound screen (Table S3). Indeed, the enrichment score of genuine PI3K/Akt/mTOR inhibitors of 12.5 (*p* = 1.03E‐29) is significantly higher than the score for the general PI3K/Akt/mTOR inhibitors (8.7, *p* = 1.17E‐19; Figure 4B).

### 3.5 mRNA Analysis Confirms Cell Cycle and PI3K/Akt/mTOR Targeting Compounds as Regulators of C/EBPδ Expression

Although we selected a C/EBPδ‐specific binding sequence to activate the eGFP reporter, other C/EBP family members may bind to this sequence with altering specificities [18]. Therefore, it cannot be excluded that other C/EBP members are modified by the compound library and subsequently modify eGFP transcription and subsequent eGFP protein levels. We therefore assessed the level of *CEBPD* mRNA upon treatment with the cell cycle and PI3K/Akt/mTOR‐targeting compounds identified above. All the cell cycle targeting compounds induced *CEBPD* mRNA expression, whereas 10 of the 12 compounds that target the PI3K/Akt/mTOR pathway did not yield valid RT‐qPCR results upon repeated attempts which might likely be a result of significantly reduced *CEBPD* mRNA by these compounds (Table S3). The other two compounds, GSK690693 and GDC‐0068, induced *CEBPD* mRNA levels despite lowering eGFP intensity suggesting that these compounds reduce expression of other proteins involved in eGFP transcription and both *CEBPA* and *CEBPB* mRNA levels were indeed reduced by GSK690693 and GDC‐0068 (not shown). Of note, except for a very small increase in *CEBPA* mRNA levels of around 25%, the cell cycle targeting compounds did not induce *CEBPA* or *CEBPB* mRNA expression.

### 3.6 Validation of C/EBPδ‐Activating Compounds in PDAC Cells

**FIGURE 5:**
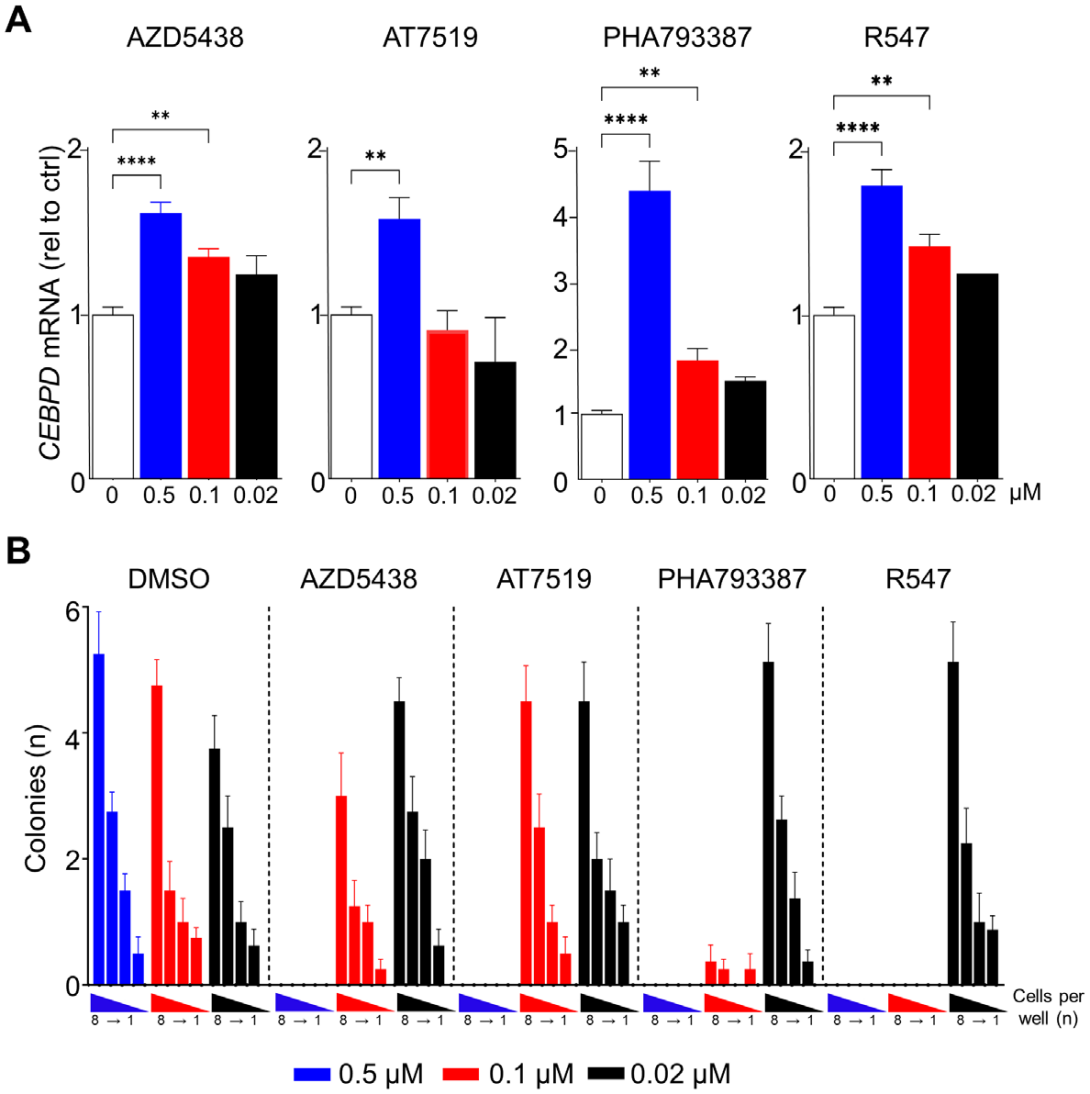
Validation of C/EBPδ‐activating compounds in PDAC cells. (A) CEBPD mRNA expression in PANC1 cells after treatment with the indicated. Shown is the mean ± SEM (*n* = 3) at t = 72 h. ****p < 0.0001; **p < 0.01. (B) C/EBPδ‐inducing compounds inhibit clonogenicity in PANC‐1 cells. Shown is the number of colonies (mean ± SEM; *n* = 8) observed 3 weeks after seeding 8, 4, 2 or 1 cell(s)/well in the presence of the indicated compounds. DMSO (solvent) was used as negative, no treatment, control.

This compound screen is of potential interest in the context of different diseases where C/EBPδ plays an important role, and the compounds that inhibit C/EBPδ activity may hold promise in lung adenocarcinoma, glioma and urothelial bladder cancer in which C/EBPδ is overexpressed and associates with poor survival [4, 5, 6]. We are however particularly interested in the identification of compounds that activate C/EBPδ in PDAC cells. This is based on the notion that C/EBPδ expression is lost in PDAC cells, that this loss correlates with shorter overall survival in PDAC patients and that C/EBPδ re‐expression in PANC1 PDAC cells dramatically reduced their clonogenic capacity [15]. Consequently, we next assessed the effect of a selection of C/EBPδ‐inducing compounds on *CEBPD* mRNA levels in PANC1 cells. We focused on the compounds R547, AT7519, AZD5438 and PHA793387 based on their ability to strongly and repeatedly induce eGFP reporter fluorescence, to upregulate *CEBPD* mRNA levels in HEK 293 T cells, and based on comparably low observed cytotoxicity in HEK 293 T cells (Table 1, Table S3). As shown in Figure 5A, all drugs induced *CEBPD* mRNA levels in a concentration‐dependent manner with PHA793387 showing the highest induction. Finally, we determined the effect of the selected drugs on the clonogenic capacity of PANC‐1 cells. To this end, one to eight cells were seeded into 96‐well plates after which colony formation was observed over time in the presence of the different C/EBPδ‐inducing compounds. As shown in Figure 5B, we observed a significant reduction in clonal outgrowth by all drugs at the 0.5 μM concentration, whereas R547 and PHA793387 also limited colony formation at 0.1 μM. None of the compounds affected clonal outgrowth at the low 0.02 μM concentration that also did not induce *CEBPD* mRNA levels in PANC1 cells (Figure 5A).

## 4 Discussion

C/EBPδ is a transcription factor that plays a key role in differentiation‐associated processes such as cell cycle regulation, proliferation and apoptosis in healthy tissue development and during cancer progression [1, 2, 3]. Interestingly, C/EBPδ may either act in a tumour‐specific manner as a tumour promotor [4, 5, 6] or tumour suppressor [7, 8, 9, 10, 11, 12, 13, 14, 15] and C/EBPδ activity may thus need to be inhibited or enhanced to achieve (pre)clinical relevance. The lack of pharmacological C/EBPδ‐targeting compounds does, however, obscure preclinical assessment of C/EBPδ as treatment modality in cancer, and consequently, we here set out a high‐throughput screen to identify small molecules that may either inhibit or enhance C/EBPδ activity.

The tumour suppressor role of C/EBPδ and potential (pre)clinical relevance of C/EBPδ reactivation is most evident in PDAC. Indeed, C/EBPδ is strongly suppressed in the nuclei of PDAC cells and its loss is associated with decreased patient survival and an enhanced likelihood of lymph node involvement [15]. Re‐expression of C/EBPδ from exogenous overexpression constructs results in decreased proliferation, migration and clonogenicity of PANC‐1 and MIA PaCa‐2 PDAC cells [15, 20]. The exact mechanism by which C/EBPδ exerts these cellular responses remains elusive although we previously hypothesised that retarding effects of C/EBPδ on the actin cytoskeleton may at least in part be responsible for the observed growth inhibitory effects on PDAC cells [21]. Irrespective the actual mechanism, these insights suggest that C/EBPδ acts as a tumour suppressor in the context of PDAC and that its reactivation could positively influence clinical outcomes.

To date only few compounds for the activation of C/EBPδ have been discovered in individual studies. Those include 1‐(2‐hydroxy‐5‐methylphenyl)‐3‐phenyl‐1,3‐propanedione (HMDB) in epidermoid carcinoma xenografts [22], all‐trans retinoic acid in acute myeloid leukaemia cells [23], anti‐cancer drugs including 5‐fluorouracil and cisplatin in fibroblasts [24], lipopolysaccharide (LPS) in the (lung) inflammatory response [25], metformin in hepatocellular carcinoma [26] and propranolol in the context of haemangioma [27]. Furthermore, a high‐throughput drug screen to identify compounds modulating C/EBPδ in the context of inflammation using LPS‐ and IFN‐γ‐activated pro‐inflammatory M1 macrophages revealed two HDAC inhibitors—Trichostatin A and Vorinostat (SAHA, MK0683)—as well as two BET inhibitors—Ro 11‐1464 and I‐BET151 (GSK1210151A)—that resulted in an activation of C/EBPδ‐mediated reporter activity [28]. While the latter confirms the observations made in our eGFP reporter screen, albeit using different BET inhibitors (CPI‐203 and (+)‐JQ1), BET inhibition induced *CEBDP* mRNA in macrophages but decreased its levels in HEK 293 T cells. Potential explanations might lie in the specificity of different BET proteins for *CEBPD*‐transcribing factors and C/EBPδ itself. Yet another study found that, in TNF‐α‐treated vascular smooth muscle cells, C/EBPδ is epigenetically controlled by BET family member bromodomain protein 4 (BRD4) and that BRD4 inhibition decreased *CEBPD* mRNA levels [29]. The inconsistency among different model systems emphasises the variety of mechanisms through which C/EBPδ is (de‐)regulated in different cell types.

Using a high‐throughput screen of 1402 small molecule inhibitors and a eGFP reporter cell line responsive to C/EBPδ transcriptional activity, we here identified further C/EBPδ‐modulating compounds and complement the list of C/EBPδ‐inducing compounds with four small molecule inhibitors which are particularly effective in PDAC cells. Those compounds are the ATP‐competitive cell cycle inhibitors R547, AT7519, AZD5438 and PHA793387, all targeting CDK1 and 2, while PHA793387 additionally targets CDK5 and 7 whereas AT7519 and AZD5438 also target CDK4 and 9. CDKs are kinases required for the progression of the cell cycle through its main phases G1, S, G2 and M‐phase. According to their role in cell cycle regulation, the inhibitors have evoked growth inhibition in different cancer models [30, 31, 32].

Generally, an induction of C/EBPδ upon halting of the cell cycle is in agreement with its role in differentiation, which occurs to a large extend in non‐ or slowly proliferating cells. To date, however, the literature has largely been concerned with C/EBPδ‐mediated regulation of the cell cycle rather than vice versa [33, 34]. Yet, a role of cell cycle regulatory mechanisms in the expression and activation of C/EBPδ is beginning to emerge; CDK1, 2, 3, 4, 6 and 7 are the main CDKs involved in cell cycle progression in human cells. To initiate transition from the quiescent G0 towards the G1 phase, the Cyclin D‐CDK4/6 complex partially phosphorylates Retinoblastoma (Rb) to release E2F transcription factors, allowing transcription of G1‐specific genes [35]. Inhibition of CDK4/6 consequently keeps cells in a G0 growth arrest. In mouse mammary epithelial cells, C/EBPδ is induced during G0 growth arrest in response to serum withdrawal [36]. While the underlying pathways of C/EBPδ induction during G0 has not been uncovered yet, a putative mechanism of CDK inhibitor‐mediated C/EBPδ activation can be derived from work showing that transcription of *CEBPD* is continuously hampered by hypermethylation through the H3K27 methyltransferase Enhancer of Zeste Homologue 2 (EZH2) in cervical cancer and hepatocellular carcinoma [10]. Of note, EZH2 expression lies downstream of the above‐described Rb/E2F pathway [37]. Decreased CDK4/6 activity thus reduces phosphorylation of Rb, subsequent E2F‐release and EZH2 induction to allow demethylation and transcription of *CEBPD*. Interestingly, EZH2 is specifically expressed in growing cells [37] which is again in line with the finding that C/EBPδ correlates with growth arrest.

Opposing to its role in PDAC, C/EBPδ is known to act as a tumour promoter in other cancers including glioblastoma [38] and urothelial bladder cancer [6]. In such contexts, and specifically where C/EBPδ contributes to drug resistance and hypoxia adaption [3], it might be beneficial to suppress C/EBPδ expression and activity. Similarly, C/EBPδ is involved in the acute inflammatory response [39, 40], where a negative regulation of its activity might thus have beneficial effects. To facilitate such approaches, this study reveals 18 non‐cytotoxic compounds for the suppression of C/EBPδ (Table 1). Interestingly, among those, the compounds targeting the PI3K/Akt/mTOR pathway are most significantly enriched. A putative explanation for this effect might lie not too far downstream of this pathway, in the Akt‐mediated phosphorylation of the cyclic adenosine monophosphate (cAMP) responsive element‐binding protein (CREB). Together with CREB‐binding protein, the CREB transcription factor binds to cAMP response elements in the *CEBPD* promoter to initiate its transcription [41, 42, 43]. Next to nuclear factor‐kappa B (NF‐kB) and signal transducer and activator of transcription 3 (STAT3), CREB is one of the most important inducers of C/EBPδ [28]. Inhibition of CREB's upstream activating pathway thus likely reduces the expression and subsequent activity of C/EBPδ. Before pursuing one of the 18 identified compounds that suppress C/EBPδ in, for instance, glioblastoma, urothelial bladder cancer or the acute inflammatory response, one should realise that compounds modifying C/EBPδ in HEK cells may also not regulate C/EBPδ in other cell types. It will thus be pivotal to first assess whether the identified compounds indeed reduce C/EBPδ activity in relevant cell types driving these disorders.

Although we validated the effectiveness of the most potent C/EBPδ‐inducing compounds in PDAC clonogenicity assays, one must consider that this study is not free of limitations. Two hundred twenty‐eight compounds were excluded from further analysis due to cytotoxicity in the first screen. Importantly, although C/EBPδ overexpression did not induce cytotoxicity in HEK 293 T (Figure 1), we cannot rule out that these compounds induce C/EBPδ to such high levels that C/EBPδ induces cell killing—a desirable effect in PDAC. Alternatively, these drugs might have C/EBPδ‐modulating properties at concentrations below 1 μM without being cytotoxic. Conversely, compounds without measurable effects at the applied concentration of 1 μM might require a higher concentration to effectively modulate C/EBPδ. Altogether, it cannot be excluded that these groups contain compounds with valuable properties that were missed due to the set‐up of this screen. Furthermore, we studied C/EBPδ activity in HEK 293 T cells, using eGFP reporter expression as a high‐throughput readout, followed by assessment of *CEBPD* mRNA levels. Despite the correlation between eGFP and *CEBPD* mRNA levels, our study cannot rule out that C/EBP proteins other than C/EBPδ are modulated by CDK inhibition and that these C/EBP proteins may have influenced eGFP levels. CDK2 for instance has been shown to modulate the activity of the closely related C/EBPβ through phosphorylation at Ser64 which would naturally also be affected by CDK2 inhibition [44]. Such effects might after all bias the interpretation of the eGFP reporter signal and must be anticipated in a disease‐ or tissue‐specific manner when using any of the CDK targeting compounds for C/EBPδ activation. Another potential limitation of our study may be the lack of in vivo experiments as our in vitro experiments obviously do not accurately recapitulate the complex physiological and pathological conditions in living organisms. Finally, one could argue that the lack of insight into the mechanism by which the identified compounds modify C/EBPδ activity is a potential limitation. Indeed, the compounds could modulate C/EBPδ activity by acting on *CEBPD* transcription but also on post‐translational modifications that could (for instance) modify dimerisation or nuclear translocation of C/EBPδ thereby increasing its transcriptional activity. Deducing the actual mechanism by which the studied drugs modify the transcriptional activity of C/EBPδ is of obvious interest but is not directly the topic of this research paper and the lack of such experiments do not take away anything of the message of our paper.

Although we have identified different compounds that modulate the activity of C/EBPδ, none of these molecules target C/EBPδ directly. Instead, they modulate overarching pathways that not only induce C/EBPδ activity but also non‐specifically modulates the expression of multiple downstream genes likely with limited functional commonality. Although of scientific interest, clinically this may pose limitations as such an approach expected to result in off‐target effects with consequent dose‐limiting toxicities [45]. Alternative approaches for direct targeting, that is, suppression, of C/EBPδ are currently being investigated. Cell‐penetrating C/EBPδ leucine zipper mimics were, for instance, able to prevent the formation of transcriptionally active C/EBPδ dimers and induced apoptosis in multiple cancer cell lines [46]. Similarly, engineered dominant‐negative (DN) binding partners of C/EBPδ, such as DN‐ATF5 lacking a DNA‐binding domain, have been used to sequester C/EBPδ (and C/EBPβ) monomers which again promoted apoptotic cell death [47]. Means for the targeted activation of C/EBPδ are still lacking. Irrespective of these limitations, this study has given implications on the cell cycle‐mediated regulation of C/EBPδ and yields a valuable selection of C/EBPδ‐modulating compounds for use in preclinical studies.

## 5 Conclusions

We have probed 1402 compounds for their effect on C/EBPδ transcriptional activity in HEK 293 T cells. A high‐throughput eGFP‐reporter assay revealed a list of 22 compounds that reproducibly induce C/EBPδ‐mediated eGFP reporter activity and 18 compounds that suppress C/EBPδ activity. Specifically the CDK‐inhibitors R547, AT7519, AZD5438 and PHA793387 strongly induced C/EBPδ activity and mRNA in HEK 293T cells which we confirmed in PDAC cells. Functionally, R547, AT7519, AZD5438 and PHA793387 all inhibited PDAC clonogenicity in vitro suggesting these compounds may be used to re‐activate C/EBPδ in preclinical studies to assess potential clinical benefits.

## Author Contributions

**Leonie Hartl:** conceptualization (equal), data curation (lead), formal analysis (lead), investigation (lead), methodology (lead), validation (lead), visualization (lead), writing – original draft (lead), writing – review and editing (equal). **JanWillem Duitman:** conceptualization (equal), methodology (equal), resources (equal), supervision (equal), validation (supporting), writing – review and editing (equal). **Hella L. Aberson:** data curation (supporting), investigation (supporting), methodology (supporting), validation (supporting). **Jan Paul Medema:** methodology (supporting), resources (supporting), writing – review and editing (supporting). **Maarten F. Bijlsma:** conceptualization (equal), methodology (equal), resources (equal), supervision (equal), validation (equal), writing – review and editing (equal). **C. Arnold Spek:** conceptualization (equal), data curation (equal), formal analysis (supporting), funding acquisition (lead), investigation (supporting), methodology (equal), project administration (lead), resources (equal), supervision (lead), validation (supporting), visualization (supporting), writing – review and editing (equal).

## Conflicts of Interest

M.F.B. has received research funding from Celgene and Lead Pharma and has acted as a consultant for Servier and Olympus. None were involved in the design of this study or drafting of the manuscript. All other authors declare no conflict of interest.

## Supporting information


**Figure S1** Effect of small molecule compounds on C/EBPδ‐induced eGFP expression. Plotted are all 1402 compounds from the library (mean of run 1 and run 2) categorized by the pathway the small compounds are targeting. Dotted lines show the mean ± 1 SD from the first run based upon which compounds for the second run were selected (i.e. those drugs with normalized eGFP levels < mean − 1 SD or > mean + 1 SD).
**Table S1** Primers used for RT‐qPCR analysis.
**Table S2** Overview of compounds that repeatedly modify C/EBPδ activity.
**Table S3** Compounds from the cell cycle pathway that induces C/EBPδ activity and from the PI3K/Akt/mTOR pathway that inhibits C/EBPδ activity.

## Data Availability

All relevant data are within the paper and its supporting information files.
