# Supplementary material for: Identification of C/EBPδ‐Modifying Compounds as Potential Anticancer Agents Using a High‐Throughput Drug Screen
Source: J Cell Mol Med. 2025 Jan 31;29(3):e70287. doi: 10.1111/jcmm.70287 (PMC11783153; doi:10.1111/jcmm.70287)
Supplement: Supplementary file 1 — Figure S1 Effect of small molecule compounds on C/EBPδ‐induced eGFP expression. Plotted are all 1402 compounds from the library (mean of run 1 and run 2) categorized by the pathway the small compounds are targeting. Dotted lines show the mean ± 1 SD from the first run based upon which compounds for the second run were selected (i.e. those drugs with normalized eGFP levels < mean − 1 SD or > mean + 1 SD). Table S1 Primers used for RT‐qPCR analysis. Table S2 Overview of compounds that repeatedly modify C/EBPδ activity. Table S3 Compounds from the cell cycle pathway that induces C/EBPδ activity and from the PI3K/Akt/mTOR pathway that inhibits C/EBPδ activity. [file JCMM-29-e70287-s001.pdf]

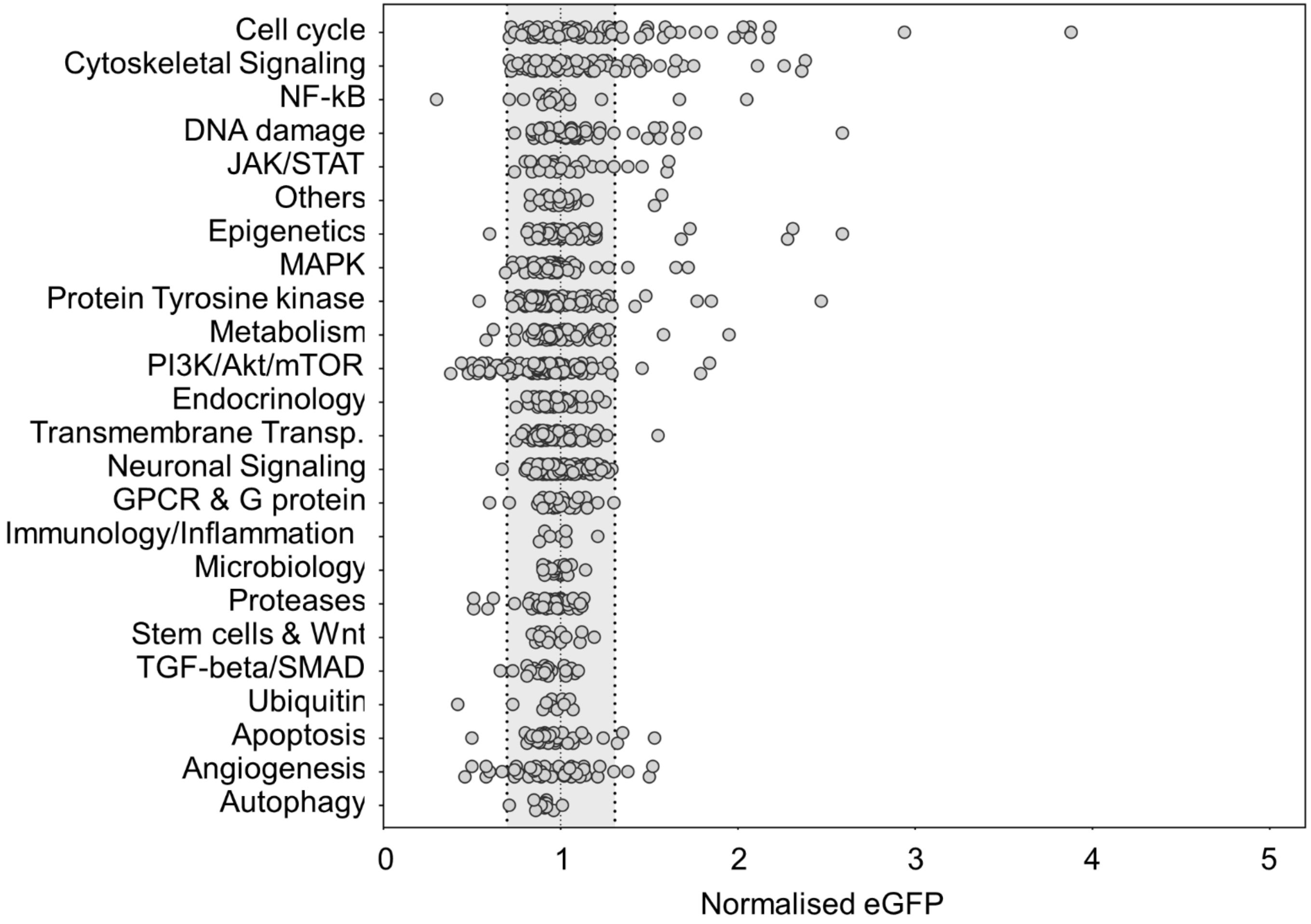

Figure S1. Effect of small molecule compounds on C/EBPδ-induced eGFP expression. Plotted are all 1402 compounds from the library (mean of run 1 and run 2) categorized by the pathway the small compounds are targeting. Dotted lines show the mean ± 1 SD from the first run based upon which compounds for the second run were selected (i.e. those drugs with normalized eGFP levels < mean – 1 SD or > mean + 1 SD).

Table S1. Primers used for RT-qPCR analysis.

Gene	Primer Sequence
TBP forward	5'-ATCCCAAGCGGTTTGCTGC-3
TBP reverse	5'-ACTGTTCTTCACTCTTGGCTC-3'
RPLP0 forward	5'-GGCACCATTGAAATCCTGAGTGATGTG-3'
RPLP0 reverse	5'-TTGCGGACACCCTCCAGGAAGC-3'
CEBPD forward	5'-GCAGAAGTTGGTGGAGCTGT-3'
CEBPD reverse	5'-TTACCGGCAGTCTGCTGTC-3'

Table S2. Compounds that repeatedly modify C/EBPδ activity. Shown is the fold eGFP expression of the 1 µM concentration in the (1st) exploration run and the 2nd validation run to the DMSO-normalized mean. Compounds are ordered based on the average eGFP expression and compounds that induced / inhibited C/EBPδ activity towards ≥ 1 SD above or below the mean, respectively, are numbered based on their average effect.

Number	Compound	Target	Pathway	1st Run	2nd Run	No. up/down
**Inducing compounds**						> 1 SD
1	R547	CDK	Cell Cycle	5.41	2.36	1
2	OTX015	BET	Epigenetics	2.90	2.29	2
3	Daunorubicin HCl	Topoisomerase	DNA Damage	2.53	2.64	3
4	GNF-5837	Trk receptor	Protein Tyrosine Kinase	2.96	1.97	4
5	PCI-24781 (Abexinostat)	HDAC	Cytoskeletal Signaling	1.92	2.85	5
6	M344	HDAC	Cytoskeletal Signaling	2.13	2.59	6
7	(+)-JQ1	BET	Epigenetics	2.44	2.19	7
8	CPI-203	Epigenetic Reader Domain	Epigenetics	2.51	2.05	8
9	Belinostat (PXD101)	HDAC	Cytoskeletal Signaling	1.82	2.71	9
10	TAK-901	Aurora Kinase	Cell Cycle	2.95	1.42	10
11	AT7519 HCl	CDK	Cell Cycle	2.20	2.14	11
12	Pracinostat (SB939)	HDAC	Cytoskeletal Signaling	2.13	2.08	12
13	PHA-793887	CDK	Cell Cycle	2.25	1.89	13
14	Dinaciclib (SCH727965)	CDK	Cell Cycle	1.37	2.76	14
15	AT7519	CDK	Cell Cycle	1.46	2.67	15
16	WS6	IκB/IKK	NF-κB	2.07	2.02	16
17	Flavopiridol HCl	CDK	Cell Cycle	1.34	2.72	17
18	AZD5438	CDK	Cell Cycle	2.39	1.67	18
19	Ro3280	PLK	Cell Cycle	2.22	1.74	19
20	Pralatrexate	DHFR	Metabolism	1.47	2.43	20
21	Tenovin-1	p53	Apoptosis	1.89	1.83	21
22	Volasertib (BI 6727)	PLK	Cell Cycle	1.87	1.84	22
23	CHIR-98014	GSK-3	PI3K/Akt/mTOR	1.42	2.16	23

24	CL-387785 (EKI-785)	EGFR	Protein Tyrosine Kinase	2.10	1.44	24
25	Triapine	DNA/RNA Synthesis	DNA Damage	1.83	1.69	25
26	JNJ-7706621	CDK, Aurora Kinase	Cell Cycle	1.67	1.84	26
27	SNX-2112 (PF-04928473)	HSP	Cytoskeletal Signaling	1.92	1.58	27
28	CUDC-101	HDAC, EGFR, HER2	Epigenetics	1.69	1.77	28
29	GSK1324726A (I-BET726)	Epigenetic Reader Domain	Epigenetics	1.67	1.79	29
30	TAK-632	Raf	MAPK	1.68	1.77	30
31	WS3	IκB/IKK	NF-κB	1.80	1.55	31
32	GSK461364	PLK	Cell Cycle	1.68	1.66	32
33	Amonafide	Topoisomerase	DNA Damage	1.42	1.92	33
34	Voreloxin (SNS-595)	Topoisomerase	DNA Damage	1.76	1.56	34
35	LMK-235	HDAC	Cytoskeletal Signaling	1.49	1.82	35
36	ERK5-IN-1	ERK	MAPK	1.60	1.70	36
37	PF-04929113 (SNX-5422)	HSP	Cytoskeletal Signaling	1.80	1.48	37
38	TG003	CDK	Cell Cycle	2.17	1.07	
39	Pacritinib (SB1518)	JAK	JAK/STAT	1.67	1.56	38
40	LY2784544	JAK	JAK/STAT	1.94	1.26	
41	BI 2536	PLK	Cell Cycle	1.36	1.81	39
42	Pravastatin sodium	HMG-CoA Reductase	Metabolism	2.12	1.03	
43	LY2835219	CDK	Cell Cycle	1.44	1.71	40
44	Irinotecan	Topoisomerase	DNA Damage	1.38	1.76	41
45	Givinostat (ITF2357)	HDAC	Cytoskeletal Signaling	1.37	1.75	42
46	AG-14361	PARP	DNA Damage	1.33	1.78	43
47	Digoxin	Sodium Channel	Transmembrane Transporters	1.34	1.77	44
48	RITA (NSC 652287)	p53	Apoptosis	1.53	1.54	45
49	TH-302	Others	Others	1.39	1.67	46
50	SN-38	Topoisomerase	DNA Damage	1.71	1.35	47
51	PF-00562271	FAK	Angiogenesis	1.59	1.44	48
52	KW-2449	Flt, Bcr-Abl, Aurora Kinase	Angiogenesis	1.88	1.11	
53	(S)-10-Hydroxycamptothecin	Topoisomerase	DNA Damage	1.63	1.35	49

54	AT9283	Bcr-Abl, JAK, Aurora Kinase	Cell Cycle	1.33	1.65	50
55	MLN8054	Aurora Kinase	Cell Cycle	1.58	1.39	51
56	ZM 447439	Aurora Kinase	Cell Cycle	1.40	1.57	52
57	PF-3758309	PAK	Cytoskeletal Signaling	1.37	1.60	53
58	AST-1306	EGFR	Protein Tyrosine Kinase	1.42	1.54	54
59	LY2090314	GSK-3	PI3K/Akt/mTOR	1.33	1.60	55
60	FLLL32	JAK	JAK/STAT	1.31	1.61	56
61	HSP990 (NVP-HSP990)	HSP (e.g. HSP90)	Cytoskeletal Signaling	1.46	1.45	57
62	Docetaxel Trihydrate	Microtubule Associated	Cytoskeletal Signaling	1.66	1.24	
63	MK-8745	Aurora Kinase	Cell Cycle	1.40	1.49	58
64	XL888	HSP (e.g. HSP90)	Cytoskeletal Signaling	1.39	1.50	59
65	4SC-202	HDAC	Cytoskeletal Signaling	1.78	1.09	
66	PD168393	EGFR	Protein Tyrosine Kinase	1.47	1.37	60
67	BMN 673	PARP	DNA Damage	1.70	1.13	
68	Stattic	STAT	JAK/STAT	1.45	1.31	61
69	RAF265 (CHIR-265)	Raf, VEGFR	MAPK	1.40	1.37	62
70	SU6656	Src	Angiogenesis	1.35	1.41	63
71	GSK923295	Kinesin	Cytoskeletal Signaling	1.33	1.39	64
72	LDC000067	CDK	Cell Cycle	1.40	1.29	
73	Purvalanol A	CDK	Cell Cycle	1.34	1.33	65
74	VER-50589	HSP (e.g. HSP90)	Cytoskeletal Signaling	1.58	1.07	
75	MPI-0479605	Kinesin	Cytoskeletal Signaling	1.39	1.22	
76	MI-2 (MALT1 inhibitor)	Malt	Others	1.51	1.07	
77	Trospium chloride	AChR	Neuronal Signaling	1.39	1.16	
78	Imatinib (STI571)	PDGFR,c-Kit, v-Abl	Protein Tyrosine Kinase	1.37	1.16	
79	ENMD-2076	Flt, Aurora Kinase, VEGFR	Angiogenesis	1.41	1.09	
80	Vinorelbine Tartrate	Microtubule Associated	Cytoskeletal Signaling	1.33	1.14	
81	GSK2656157	PERK	Apoptosis	1.38	1.09	
82	Caffeic Acid Phenethyl Ester	NF-κB	NF-κB	1.36	1.09	
83	Clomifene citrate	Estrogen Receptor	Endocrinology & Hormones	1.37	1.05	

**Inhibiting compounds**						< 1 SD
84	Omaveloxolone (RTA-408)	NF-κB	NF-κB	0.14	0.46	1
85	GSK1059615	PI3K, mTOR	PI3K/Akt/mTOR	0.31	0.44	2
86	INK 128 (MLN0128)	mTOR	PI3K/Akt/mTOR	0.36	0.52	3
87	WH-4-023	Src	Angiogenesis	0.53	0.38	4
88	GSK690693	Akt	PI3K/Akt/mTOR	0.38	0.56	5
89	PF-04691502	mTOR, PI3K, Akt	PI3K/Akt/mTOR	0.44	0.53	6
90	AZD5363	Akt	PI3K/Akt/mTOR	0.51	0.47	7
91	Gambogic Acid	Bcl-2,Caspase	Apoptosis	0.37	0.63	8
92	GDC-0980 (RG7422)	mTOR, PI3K	PI3K/Akt/mTOR	0.47	0.54	9
93	Ponatinib (AP24534)	Bcr-Abl, VEGFR, FGFR	Angiogenesis	0.51	0.50	10
94	Oprozomib (ONX 0912)	Proteasome	Proteases	0.23	0.79	
95	AZD2014	mTOR	PI3K/Akt/mTOR	0.52	0.50	11
96	PI-103	DNA-PK, PI3K, mTOR	PI3K/Akt/mTOR	0.48	0.56	12
97	KU-0063794	mTOR	PI3K/Akt/mTOR	0.51	0.54	13
98	GNE-317	PI3K	PI3K/Akt/mTOR	0.65	0.42	14
99	GDC-0068	Akt	PI3K/Akt/mTOR	0.53	0.54	15
100	PP121	DNA-PK, mTOR, PDGF	PI3K/Akt/mTOR	0.65	0.43	16
101	BGT226 (NVP-BGT226)	PI3K, mTOR	PI3K/Akt/mTOR	0.62	0.51	17
102	Deltarasin	PDE	Metabolism	0.29	0.86	
103	Bosutinib (SKI-606)	Src	Angiogenesis	0.70	0.47	18
104	GZD824	Bcr-Abl	Angiogenesis	0.70	0.47	19
105	Rapamycin (Sirolimus)	mTOR	PI3K/Akt/mTOR	0.54	0.63	20
106	ONX-0914 (PR-957)	Proteasome	Proteases	0.36	0.82	
107	Dasatinib	Src, Bcr-Abl, c-Kit	Angiogenesis	0.69	0.50	21
108	PP242	mTOR	PI3K/Akt/mTOR	0.69	0.50	22
109	Temsirolimus	mTOR	PI3K/Akt/mTOR	0.52	0.68	23
110	ZSTK474	PI3K	PI3K/Akt/mTOR	0.54	0.66	24
111	SGC-CBP30	Epigenetic Reader Domain	Epigenetics	0.53	0.67	25
112	MG-132	Proteasome	Proteases	0.34	0.91	

113	Darapladib (SB-480848)	Phospholipase (e.g. PLA)	Metabolism	0.33	0.92	
114	WYE-354	mTOR	PI3K/Akt/mTOR	0.62	0.64	26
115	AZD8055	mTOR	PI3K/Akt/mTOR	0.33	0.95	
116	GDC-0941	PI3K	PI3K/Akt/mTOR	0.67	0.63	27
117	SB525334	TGF-beta/Smad	TGF-beta/Smad	0.68	0.64	28
118	Sorafenib Tosylate	VEGFR, PDGFR, Raf	MAPK	0.62	0.72	
119	Pexmetinib (ARRY-614)	p38 MAPK	MAPK	0.69	0.69	29
120	Everolimus (RAD001)	mTOR	Others	0.55	0.86	
121	IMD 0354	IKK	NF-κB	0.54	0.89	
122	MK-2206 2HCl	Akt	PI3K/Akt/mTOR	0.67	0.76	
123	NMS-873	p97	Ubiquitin	0.65	0.81	
124	Staurosporine	PKC	TGF-beta/Smad	0.56	0.91	
125	JNJ-1661010	FAAH	Metabolism	0.66	0.82	
126	Linifanib (ABT-869)	PDGFR, VEGFR	Protein Tyrosine Kinase	0.65	0.87	
127	PTC-209	BMI-1	Cell Cycle	0.61	0.94	
128	Rigosertib (ON-01910)	PLK	Cell Cycle	0.68	0.95	

Table S3. Compounds from the cell cycle pathway that induce C/EBPδ activity and from the PI3K pathway that inhibit C/EBPδ activity. Shown is the fold eGFP expression of the 1 µM concentration to the DMSO-normalized mean. Compounds are ordered based on the average eGFP expression and compounds selected for further analysis are depicted in bold. ND: not detected.

**Pathway**	**Compound**	**Target**	**eGFP**	**CEBPD mRNA**
Cell cycle	R547	CDK	3.88	↑
Cell cycle	PHA-793887	CDK	2.07	↑
Cell cycle	AT7519	CDK	2.06	↑
Cell cycle	AZD5438	CDK	2.03	↑
Cell cycle	JNJ-7706621	CDK, Aurora Kinase	1.76	↑
Cell cycle	MLN8054	Aurora Kinase	1.49	↑
Cell cycle	MK-8745	Aurora Kinase	1.45	↑
PI3K/Akt/mTOR	GDC-0941	PI3K	0.65	↑
PI3K/Akt/mTOR	WYE-354	mTOR	0.63	ND
PI3K/Akt/mTOR	ZSTK474	PI3K	0.60	ND
PI3K/Akt/mTOR	Temsirolimus	mTOR	0.60	ND
PI3K/Akt/mTOR	PP242	mTOR	0.60	ND
PI3K/Akt/mTOR	Rapamycin	mTOR	0.59	ND
PI3K/Akt/mTOR	GDC-0068	Akt	0.54	ND
PI3K/Akt/mTOR	KU-0063794	mTOR	0.53	ND
PI3K/Akt/mTOR	AZD5363	Akt	0.49	ND
PI3K/Akt/mTOR	GSK690693	Akt	0.47	↑
PI3K/Akt/mTOR	INK 128 (MLN0128)	mTOR	0.44	ND
PI3K/Akt/mTOR	GSK1059615	PI3K, mTOR	0.38	ND
